# Editorial for the Special Issue on Nanostructure Based Sensors for Gas Sensing: from Devices to Systems

**DOI:** 10.3390/mi10090591

**Published:** 2019-09-09

**Authors:** Nicola Donato, Sabrina Grassini

**Affiliations:** 1 Department of Engineering, University of Messina, 98122 Messina, Italy; 2 Department of Applied Science and Technology Politecnico di Torino, 10129 Torino, Italy

The development of solid state gas sensors based on microtransducers and nanostructured sensing materials is the key point in the design of new portable measurement systems with sensing and identification performances comparable with those of most sophisticated analytical techniques. In such a context, a lot of effort must be spent of course in the development of the sensing material, but also in the choice of the transducer mechanism and structure, in the electrical characterization of the sensor prototypes, as well as in the design of suitable measurement setups. 

After a careful peer review, seven manuscripts covering all the aspects of the sensor world were accepted for publication in this special issue. Papers [1,2] deal with sensing material preparation and the characterization of the chemico-physical and sensing properties, while further studies report about the investigation of sensing performance towards different operating conditions [3] and the optimization of the transduction mechanism and of the device package [4]. Furthermore, there are three papers focused on gas sensor systems and their application in environmental monitoring [5,6] and in the biomedical field [7].

In more detail, Xu and co-authors describe a route to fabricate gold nanoparticles (less than 20 nm in diameter) wrapped with a controllable ultrathin carbon layer (Au@C, 0.6–2 nm thick) by one step laser ablation of the noble metal target in toluene–ethanol mixed solutions. The developed sensing material was tested for the detection of low concentrations of H_2_S gas, ranging from 1 to 5 ppm, at room temperature [1]. 

Li et al. present the electrospray process to deposit ZnO patterns for gas sensing, paying particular attention on the effects of different experimental parameters on the jet characteristics and on the final properties of electrosprayed patterns. Sensing performance towards alcohol vapors are also well discussed [2]. 

Bonaccorsi et al. show how UV irradiation can improve the response of an indium oxide (In_2_O_3_) resistive sensor to detect carbon monoxide, operating at low temperature in the range of 25–150 °C. In particular, the best balance between operating temperature and UV irradiation toward low CO concentration values (from 1 to 10 ppm) was observed at 100 °C [3]. 

Considering the investigation of transduction mechanism and packaging steps, Yildiz et al. present the fabrication and packaging of a capacitive micromachined ultrasonic transducer (CMUT) using anodically bondable low temperature co-fired ceramic (LTCC). The authors point out the attention on a promising approach for high density CMUT array fabrication and the indirect integration of CMUT-IC for a miniature size packaging [4]. 

Micromachining technology is the new frontier in the realization of miniaturized systems; as a matter of fact, Jianhai Sun et al. developed a mini monitoring system integrated with a microfabricated metal oxide array sensor and a micro packed gas chromatographic (GC) column for detecting environmental gases [5]. By using the chromatographic separation capability, the MOS array sensor was able to detect each component, avoiding the technical bottleneck of mutual interference among different gases. 

Mao et al. present a set of hardware platforms to improve the efficiency of new developed E-nose; the proposed system includes a gas-sensing, film-parallel, synthesis platform, a high-throughput gas sensing unmanned testing platform, and a handheld E-nose system. The sensor arrays are produced by inkjet printing, tailoring the devices for the specific application [6]. 

Ultimately, in the biomedical field, the design and development of mini-invasive systems for gas monitoring is a real challenge. In such a scenario, breath analysis is one of the best candidates, so Gatty et al. developed and characterized an integrated amperometric sensor [7] in order to determine the hydrogen sulphide (H_2_S) concentration, one of the main reasons of malodour, in oral breath. 

We hope that this special issue gives the reader new points of view in gas sensing and miniaturized systems, taking into account their fundamental role in environmental safety and human health. The special issue wants to highlight the importance of synergy among micromachining, instrumentation and measurement, chemistry and material science to face needs and challenges in gas sensor design and development.

We would like to take this opportunity to thank all the authors for submitting their papers to this special issue. We would like to thank also all reviewers for their efforts and comments to improve the quality of the submitted papers. 
